# 2668. Real-World Test-of-Cure Practices for Oropharyngeal Gonorrhea in People with HIV

**DOI:** 10.1093/ofid/ofad500.2279

**Published:** 2023-11-27

**Authors:** Darcy Wooten, Eddie Hill

**Affiliations:** University of California, San Diego, San Diego, CA; University of California San Diego, San Diego, California

## Abstract

**Background:**

Most reported ceftriaxone-based treatment failures of gonorrhea occur in cases of oropharyngeal (OP) gonorrhea. Consequently, the CDC now recommends a test-of-cure (TOC) 7-14 days after initial treatment of OP gonorrhea. There is a lack of data on adherence to these guidelines. The purpose of this study was to examine the repeat testing practices for people with HIV (PWH) who were diagnosed with and treated for OP gonorrhea.

**Methods:**

We conducted a retrospective cohort study at the University of California at San Diego among PWH who tested positive for OP gonorrhea between 12/01/2020 and 09/05/2022. We measured the time elapsed between treatment of OP gonorrhea, ordering of repeat testing, and completion of repeat testing. We calculated the proportion of patients who had a repeat test completed within the guideline-recommended window of 7-14 days. We also measured the following secondary outcomes: 1) the rate of repeat tests that showed persistent positivity, and 2) the repeat testing completion rate after the roll-out of a home self-testing program on 01/31/2022.

**Results:**

We identified 223 PWH who tested positive for OP gonorrhea. 10.3% (23/223) of patients completed repeat testing 7-14 days after treatment. The mean duration between treatment and repeat testing was 74.2 days (range 0-530 days). 4.3% (1/23) of patients who completed repeat testing within 7-14-days of treatment and 7.5% (15/200) of patients who completed repeat testing after this period showed persistent positivity (P-value 0.58). There was no difference in adherence to TOC guideline recommendations before versus after the implementation of the home self-testing program (13.3%, 14/105 versus 8.9%, 7/79; P-value 0.16).

Management Care Cascade of Oropharyngeal (OP) Gonorrhea in People with HIV (PWH)

**Figure:**
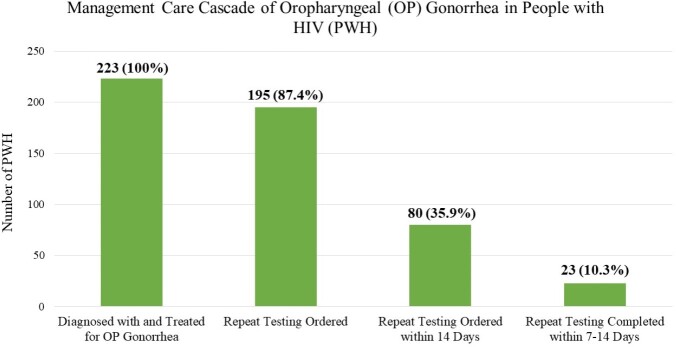

**Conclusion:**

We found that just one tenth of patients completed repeat testing 7-14 days after receiving treatment for OP gonorrhea. The rate of guideline-recommended repeat testing did not improve with the implementation of home self-testing kits for OP gonorrhea. These findings are concerning given the rising rates of antimicrobial-resistant gonorrhea and the impact that gonorrhea has on facilitating HIV transmission. Additional studies are needed to identify and understand the barriers impacting adherence to the CDC’s TOC recommendations.

**Disclosures:**

**All Authors**: No reported disclosures

